# Pre Conference Workshops And Opening Keynote Address, June 6

**DOI:** 10.3402/ejpt.v4i0.21270

**Published:** 2013-06-04

## XIII ESTSS CONFERENCE: “Trauma and its clinical pathways: PTSD and beyond”, Bologna, June 2013   PRE CONFERENCE WORKSHOPS AND OPENING KEYNOTE ADDRESS, JUNE 6

## Morning *Half Day Workshops*

### Functional and structural neuroimaging and EEG monitoring related to EMDR and CBT treatments for PTSD—EMDR workshop

#### M. Pagani Institute of Cognitive Sciences and Technologies, CNR, Rome, Italy

In the recent past, several neuroimaging studies aimed at evaluating the neural correlates of PTSD-related psychotherapies revealing their neurobiological effects on brain function. Functional studies by single-photon emission computed tomography (SPECT) and electroencephalography (EEG) detected changes in cerebral blood flow and neuronal activation patterns, identifying the brain areas implicated in the various components of emotional processing and/or affected by the disorder. Investigations by magnetic resonance imaging (MRI) have also revealed PTSD-related structural changes. The first part of the workshop will review the neuroimaging methodologies and findings in PTSD treatment-related research, with an extensive review of previous literature on the neurobiological effects of the various psychotherapies. The second part will deal with the description and implementation in research and clinic of neuropsychological testing with brief comments and discussion about their use in recent studies published by our group. In the third part the EEG monitoring of a complete set of Eye Movement Desensitization and Reprocessing therapies in 30 patients suffering of major trauma as compared to 20 healthy controls will be presented. These findings will also be compared to the neurobiological effects of trauma-focused cognitive behavioral therapy in a second group of psychologically traumatized clients. The results are the first report ever on the neurobiological changes occurring before, during and after PTSD-related psychotherapies shedding light on the neuronal processes underlying their clinical efficacy. The description and the discussion about the contents of the workshop will provide the audience (1) the necessary information to understand the methodological principles behind neuroimaging techniques (SPECT, EEG and MRI), and their possible applications in research and clinic; (2) the up-dated critical knowledge of the published papers in the field of PTSD-related psychotherapies functional and anatomical studies; (3) the basic research principles and examples to be motivated to start, take part and/or collaborate to functional studies in order to better understand the neural basis of psychotherapeutic techniques. The presented material will represent the state-of-the-art of the current neuroscience PTSD-related research and neuroimaging methodologies available at the moment.

### Brief Eclectic Psychotherapy for PTSD

#### B. Gersons^1,2^ and M. J. Mink-Nijdam^1^

^1^Department of Psychiatry, Academic Medical Center, University of Amsterdam, The Netherlands; ^2^Arq Psychotrauma Expert Group, The Netherlands

*Course objective*: Introduction to understand the framework of this effective treatment for PTSD—To understand and become familiarized with the different modules of this treatment-protocol. *Course contents*: The 16-sessions Brief Eclectic Psychotherapy for PTSD Protocol (BEPP) was originally developed for police officers (n-300) with PTSD and proved to be effective in a randomized controlled trial (RCT). A recent RCT has shown again its effectiveness with neuroimaging and a significant decrease of the heart rate. Meanwhile, BEPP has been used with excellent results for a range of other PTSD patients, e.g., following disasters (n-1300). The treatment starts with psychoeducation on PTSD. The patient and his or her partner learn to understand the symptoms of PTSD as dysfunctional, and caused by the traumatic event. The patient will then receive 4–6 sessions of relaxation and imaginary exposure, focused on the suppressed intense emotions of sorrow. Memorabilia is used to stimulate remembrances of the traumatic event and a writing task to write a letter to someone or an institution blamed for the traumatic incident. The letter is specifically used to help to express the aggressive feelings. Most symptoms will then disappear and the patient is able to concentrate on what the impact of the trauma has been on his view of him or herself and on their world. During BEPP, there should be considerable change and a new equilibrium should be reached. This is called the “domain of meaning phase”. The treatment will end by a farewell ritual with the partner in which the letter and or mementos are burned to leave the traumatic incident behind, as a way to turn and face life and future, at the same time never to forget, but not hindering the individual anymore in their daily life. Participants can find more info on www.traumatreatment.eu. The BEPP protocol is available in English, German, Lithuanian, and Dutch.

### Stabilization techniques in preparation for trauma-focused interventions with people who are refugees or seeking asylum

#### H. Kayal, J. Gratton, J. Blumberg and E. Walsh Traumatic Stress Clinic, Camden & Islington NHS Foundation Trust, London, UK

*Intended audience*: This workshop is beneficial for any mental health practitioners working with refugees or other client groups presenting with complex PTSD. It is suitable for those only intend to do phase one stabilization work as well as for those intend to go onto providing trauma-focused interventions. *Aims*: This interactive workshop will explore treatment approaches to establish a sense of safety and stability in trauma-focused therapy preparation. *Abstract*: A phased model of treatment is recommended for the people who have experienced repeated and multiple traumas and who may still be facing ongoing stress and threat. Establishing a sense of safety and stability is the first stage of treatment before any exposure work can begin. This can be particularly challenging when treating refugees with complex PTSD presentations. Case examples of torture survivors, victims of trafficking and domestic abuse will be presented to illustrate some of the difficulties in this stage of treatment and interventions. *Learning outcomes*: The workshop will promote an understanding of:

1. Complex PTSD presentations in refugees and asylum seekers.
2. Stabilization and symptom management in preparation for trauma-focused interventions.
3. Managing dissociative flashbacks, dissociative seizures and sensory/physical flashbacks.
4. Cognitive techniques for managing shame, guilt and self blame which may be barriers to exposure work.
5. How best to work with trauma memories and when to use NET, CBT or EMDR.
6. Cultural considerations.
7. Managing vicarious traumatization and self-care.

Timeline (will be adapted depending on participants)

1. Understanding of complex posttraumatic stress disorder (assessment).
2. Cultural issues to consider throughout interventions.
3. The phased model of treatment.
4. Focus on phase one of treatment.
5. Developing a shared understanding of traumatic history with the client and how it relates to PTSD and treatment model.
6. Issues of consent and readiness, addressing any social, legal, financial or practical difficulties.
7. Identifying dissociative flashbacks, dissociative seizures and sensory/physical flashbacks.
8. Grounding techniques for managing dissociation.
9. Brief overview of techniques for managing anxiety.
10. Indicators for when trauma-focused therapy is useful and which type, as well as indicators for when it is not useful.
11. Managing self-care.

### Crossing the borders: cognitive-behavioral and psychodynamic treatment for post-traumatic stress disorder

#### L. Wittmann^1^ and N. Roberts^2^

^1^International Psychoanalytic University, Berlin, Germany; ^2^Cardiff & Vale University Health Board, UK

Available empirical evidence regarding study drop outs and response rates indicates that no single therapeutic approach sufficiently addresses all needs of all trauma survivors. This clinical pre-conference workshop aims at furthering the mutual and respectful consideration of the potential benefits and limitations of cognitive-behavioral and psychodynamic approaches in the treatment of post-traumatic stress disorder (PTSD). After an introduction into the treatment rationale and empirical evidence for cognitive-behavioral and psychodynamic PTSD treatments, both approaches will be illustrated by case presentations. Together with the workshop participants, the following questions will be discussed: What are similarities and differences between both approaches? Where are strengths, challenges, and limitations of each approach? Also, the possibility of incorporating elements of one approach into the other will be explored and directions for future research emerging from these considerations will be considered.

## Keynote Address

### PTSD as a shame disorder (18:10–19:00)

#### J. L. Herman^1,2^

^1^Harvard Medical School, Boston, MA, USA; ^2^Cambridge Hospital, Cambridge, MA, USA

Posttraumatic stress disorder is classified as an anxiety disorder in the Diagnostic and Statistical Manual of Mental Disorders of the American Psychiatric Association (DSM-IV, 1994). This formulation has led to a fruitful investigation of the neurobiology of fear. Pathological derangements of the normal, innate fear-signaling system have been demonstrated in PTSD, and effective treatments have been developed aimed at reducing pathologic fear. However, this formulation fails to capture an essential characteristic of those traumas that are of human design, especially those that are repeated over a prolonged period of time. In these cases, where a relationship of dominance and subordination is established, feelings of humiliation, degradation, and shame are central to the victim's experience. The resultant posttraumatic condition can be conceptualized both as an anxiety disorder and also as a shame disorder. This lecture will explore the central role of shame in complex trauma.

